# Continuous Ultrasonic Reactors: Design, Mechanism and Application

**DOI:** 10.3390/ma13020344

**Published:** 2020-01-11

**Authors:** Zhengya Dong, Claire Delacour, Keiran Mc Carogher, Aniket Pradip Udepurkar, Simon Kuhn

**Affiliations:** Department of Chemical Engineering, KU Leuven, 3001 Leuven, Belgium; zhengya.dong@kuleuven.be (Z.D.); claire.delacour@kuleuven.be (C.D.); keiran.mccarogher@kuleuven.be (K.M.C.); aniketpradip.udepurkar@kuleuven.be (A.P.U.)

**Keywords:** microfluidics, ultrasound, process intensification, sonochemistry, flow chemistry

## Abstract

Ultrasonic small scale flow reactors have found increasing popularity among researchers as they serve as a very useful platform for studying and controlling ultrasound mechanisms and effects. This has led to the use of these reactors for not only research purposes, but also various applications in biological, pharmaceutical and chemical processes mostly on laboratory and, in some cases, pilot scale. This review summarizes the state of the art of ultrasonic flow reactors and provides a guideline towards their design, characterization and application. Particular examples for ultrasound enhanced multiphase processes, spanning from immiscible fluid–fluid to fluid–solid systems, are provided. To conclude, challenges such as reactor efficiency and scalability are addressed.

## 1. Introduction

Small scale flow reactors, namely micro and milli-reactors, have great advantages over conventional reactors, such as well-controlled flow patterns and increased surface-to-volume ratios, resulting in enhanced heat and mass transfer rates [1,2,3,4,5,6]. Coupled with other benefits such as inherent safety allowing to perform reactions at elevated temperatures, pressures, or using highly reactive intermediates, they have become an attractive choice for the continuous manufacturing of chemicals and pharmaceuticals [7,8,9,10,11,12]. However, these appealing applications are still hindered by two important problems namely, weak convective mixing and issues regarding solid handling [13,14,15,16,17,18,19,20]. Weak convective mixing can be avoided with the use of passive mixing structures (such as bends, necks and baffles), however, these structures make reactors more susceptible to clogging [21,22,23,24,25].

**Figure 1:**
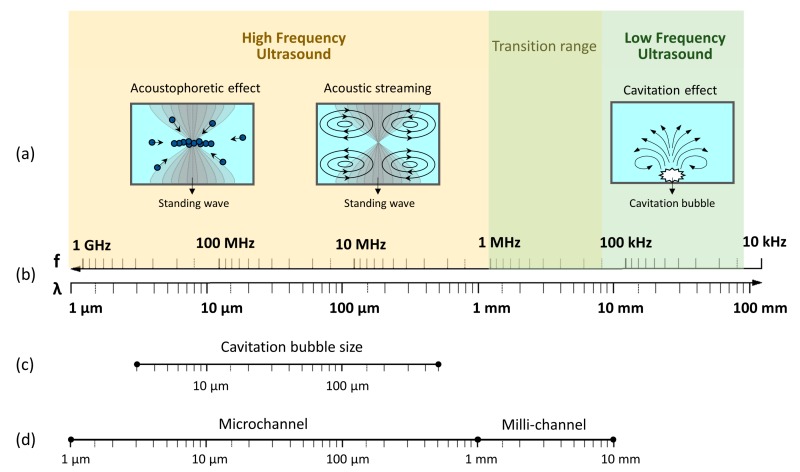
Representation of the key concepts behind ultrasonic small scale flow reactors. Firstly, (**a**) the different phenomena associated with high and low frequency ultrasound, (**b**) the ultrasonic frequency (f) and the corresponding wavelength (λ) in water, (**c**) the cavitation bubble resonance size for low frequency ultrasound (20 kHz–1 MHz) and (**d**) how the associated ultrasonic phenomena match the typical size range of micro and milli-reactor channels.

Integrating ultrasound with small scale flow reactors has proven to be one of the more promising methods to address clogging and mixing issues [13,14,15,24,26]. In fact, in batch and large scale reactors, ultrasound has been widely used to intensify mixing, mass transfer and reaction rates in various chemical and biological processes [27,28,29,30,31]. However, it is considered difficult to control and scale, due to non-uniformly generated acoustic fields and the complex flow patterns within conventional reactors [30,32,33]. Small scale reactors, on the other hand, offer a solution to these issues since the size range of ultrasonic effects are within the size range of that of the channels, see Figure 1. Therefore, the synergistic combination of them could utilizes one’s advantages to solve another’s problems [26,34,35,36].

Ultrasound is generally classified into low and high frequency ultrasound due to the different physical mechanisms that can be induced. The boundary between low and high frequency ultrasound is not necessarily strict and the transition range is typically recognized within 200 kHz and 1 MHz, as shown in Figure 1. Low frequency ultrasound generates cavitation micro-bubbles, which can intensify mixing [24,37] and interfacial mass transfer [38,39], break up agglomerates [40,41] and detach particles deposited on microchannel surfaces to prevent clogging [42,43,44,45]. Secondly, the induced cavitation bubble’s resonance size matches that of the channel, making it an ideal platform to investigate and harness cavitation effects [26,34,35,36]. High frequency ultrasound, on the other hand, is operated at power levels below the cavitation threshold, therefore cavitation effects are normally not observed. However, the wavelength in most fluids matches the channel size, making it possible to form a standing wave within the channel and utilize the associated effects, such as acoustic radiation force and streaming, see Figure 1a [46,47,48]. The radiation force is able to displace particles to pressure nodes, while the resulting acoustic streaming is able to enhance mixing [47,48,49,50]. These principles have already been successfully implemented in microreactors for acoustofluidic applications, such as cell/particle manipulation (separation, concentration and sorting) and fluid mixing for biological and chemical processes [46,51,52,53]. More recently studies show that particle manipulation using high frequency ultrasound, can also decrease solid attachment on channel walls and in turn prevent clogging [49,54].

Despite the many advantages and large number of studies on the combination of ultrasound with flow reactors, there are still a few challenges remaining, especially when it comes to scalability. These challenges include the efficient transfer of ultrasound energy from transducer to reactor as well as methods to utilize and promote ultrasonic effects, all in a bid to improve the energy efficiency of reactors. This review aims at addressing these challenges and summarizes the state of the art of ultrasonic small scale flow reactors, as well as providing a guideline to the design, characterization, application and scaling of these systems.

## 2. Physical Mechanisms of Ultrasound

The different physical mechanisms behind ultrasound are the reason for its versatility. Understanding these mechanisms lead to reactor designs that utilize the effects more efficiently and provides methods to improve reactor performance.

### 2.1. Cavitation Phenomena in Microchannels

Almost all applications of low frequency ultrasound are based on cavitation effects. When ultrasound is applied in a liquid, cavitation microbubbles are generated from gas nuclei dissolved in the liquid or trapped at the reactor wall [55,56,57]. The formation, growth, oscillation and collapse of these bubbles under the influence of the sound field is termed as acoustic cavitation [27,58,59,60,61,62]. With the increase of acoustic pressure, cavitation bubbles change from stable volume and shape oscillation to transient bubble collapse, generating liquid microstreaming, jets and shock waves. These physical effects have been widely applied to intensify mass transfer processes, such as cleaning, mixing, emulsification and extraction. On the other hand, the violent bubble collapse generates enormous temperatures and high pressure changes at a localized level, which produce radical or radical-ion intermediates that can react with reactants and thus accelerate some reactions. The oscillating intensity of cavitation bubble also depends on the bubble size and ultrasound frequency. For the frequency (f), the size of bubbles that have the strongest cavitation phenomena is usually near the linear resonance radius ($R_{r}$):

(1) $$R_{r}=\frac{1}{2\pi f}\sqrt{\frac{2\gamma P_{h}}{\rho }},$$

where $\gamma =\frac{C_{p}}{C_{v}}$ is the ratio of the specific heat of the gas at a constant pressure to its specific heat at a constant volume, ρ is the density of liquid and $P_{h}$ is the hydrostatic liquid pressure [61,63]. For air bubbles in water, the estimated resonance size is represented in Figure 1c for a frequency range of 20 kHz–1 MHz.

**Figure 2:**
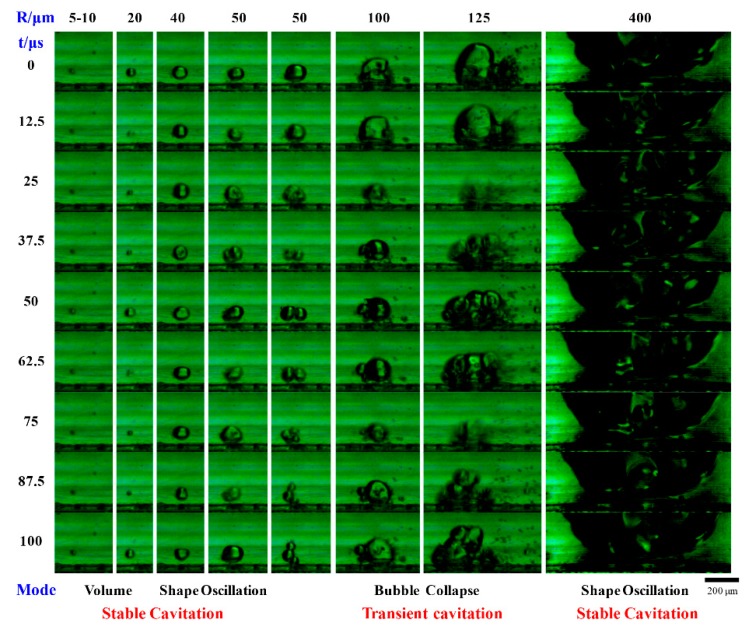
Effect of bubble radius on their cavitation behavior under ultrasound at a frequency of 20 kHz and a load power of 20 W. Bubble cavitation behavior was observed using a high-speed camera at an interval of 12.5 µs, equaling to a quarter of ultrasound oscillating period. Reprinted with permission from [24], copyright John Wiley and Sons.

Dong et al. [24] characterized the cavitation behavior of bubbles with different radii in microchannels in a ultrasound field of 20 kHz, as shown in Figure 2. With the increase of bubble size, the bubble oscillation changes from volume to shape oscillations, and finally turns into transient cavitation when the bubble radius approaches the resonance size (150 µm in water). Bubbles larger than the resonance size undergo shape oscillations with dramatic surface wave distortion, resulting in strong microstreaming around it. This cavitation microstreaming is the steady flow formed due to the dissipation of acoustic energy near an oscillating bubble, this along with the rapid motion of cavitation bubbles can result in complex flow patterns [50,64,65]. These flow patterns improve liquid mixing and accelerate gas–liquid mass transfer, which will be discussed in detail in Section 4.

An important phenomenon observed in microreactors is the confinement effect [38,66,67,68]. It was reported that cavitation phenomena in small microchannels are generally weaker than that in larger channels under the same ultrasound field. This is because smaller microchannels confine cavitation bubbles in a limited space, which produces a larger viscous resistance when bubbles oscillate. Zhao et al. [67] compared the cavitation activity of various bubbles in five square microchannels with sizes ranging from 0.5 to 2.5 mm. Cavitation activity was reduced when the channel size was decreased from 1 to 0.5 mm, while no significant difference was observed in channels larger than 1 mm. This implies that 1 mm is the critical channel size above which the confinement effect disappears. Besides the confinement effect, the hydrodynamic pressure drop in small channels is higher than in larger channels when operated at the same flow rate, this could also weaken the cavitation activity.

Due to the limited number of cavitation nuclei in the liquid, improving cavitation activity is an important topic for both continuous and batch reactor. Due to the same size range of microchannels and cavitation bubbles, artificial bubbles can be easily introduced into a microchannel [35,38,39,56,69,70]. The most common method is to fabricate micro-holes or grooves into the channel, which will trap bubbles of a specific size and initiate cavitation nuclei when ultrasound is turned on [37,69,71,72]. Tovar et al. [73,74] proposed the concept of ‘side cavity acoustic driver’, that is, chambers or grooves, processed in the sidewall of the channel that trap bubbles when the liquid enters. When ultrasound with a frequency close to the resonance frequency of the bubbles is applied, intense cavitation phenomena are generated, which then can mix and even pump fluids. Ozcelik et al. [75] found that, by fabricating a rough wavy surface into the microchannel wall, the surface initiates cavitation bubbles in the presence of acoustic waves, producing fast and effective mixing. Injecting a stream of gas bubble into the microchannel also improves cavitation activity. Tandiono et al. [76,77] investigated the effect of a 100 kHz ultrasonic field on gas–liquid slug flow in a microchannel. Upon ultrasound application, the gas–liquid interface vibrated violently, breaking up into a large number of bubble fragments, which then acted as cavitation nuclei for acoustic cavitation, producing a huge number of free radicals and intense light emissions. Dong et al. [38] found that these strong cavitation phenomena on the gas–liquid interface also accelerated the gas-liquid mass transfer significantly. This has led to the development of many applications that utilize this phenomenon, which is discussed in Section 4.

### 2.2. Standing Acoustic Waves in Microchannels: Acoustophoretic Force and Streaming

**Figure 3:**
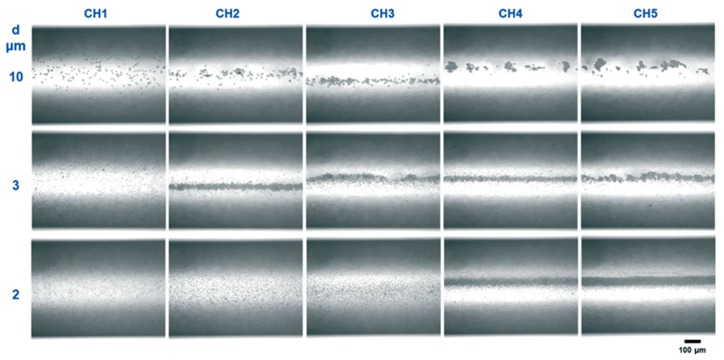
Focusing of polystyrene particles in a microchannel by high frequency ultrasound (1.21 MHz and 15 Vpp) for different particle sizes (2–10 µm). The images in each row were taken at different channel positions with the channel length and thus residence time increasing from CH1 to CH5. Reprinted with permission from [49], copyright Royal Society of Chemistry.

As mentioned earlier for high frequency ultrasound, standing waves are often formed in microchannels as the corresponding wavelength approaches that of the channel height or width. Particles in a standing wave experience acoustic radiation forces that move particles either to the pressure node or antinode, known as acoustophoresis, see Figure 1a [46,47,48,78,79,80]. Particle movement is influenced by the acoustic contrast factor [53,80,81,82,83], which is a function of fluid and particle density, compressibility and the speed of sound in the mixture. The positive or negative contrast factor results in movement of particle to the node or antinode respectively and has been applied successfully to separate particles in a suspension into two fractions [84,85,86]. The magnitude of the radiation force on particles is proportional to the particle volume, and researchers have designed microfluidic channels to separate particles of different sizes with ease [81,84,87,88,89,90]. Dong et al. [49] studied the effect of particle size on focusing effect in the microreactor and observed that bigger particles experience larger acoustophoretic force and are focused in a shorter time, see Figure 3. This acoustophoretic effect also has the potential to overcome clogging issues by focusing particles to the channel center thus avoiding particle contact with channel walls [49,54]. Dong et al. [49] have successfully demonstrated the effectiveness of standing acoustic waves to prevent clogging in a microchannel.

Another phenomenon observed with high frequency ultrasound is acoustic streaming. The two major types of streaming that can be observed as a result of standing waves are boundary layer streaming and Eckart streaming. Eckart streaming is observed for channel dimensions in the order of a few centimeters and is hence not typically found in microchannels [48,50]. Boundary layer streaming, on the other hand, which includes Schlichting and more importantly Rayleigh streaming, is the flow generated by the viscous dissipation of acoustic energy in the fluid boundary layer and is the main streaming phenomenon observed in microchannels [48,50]. Bengtsson et al. [91] used Rayleigh streaming to improve mixing in microchannels. They also noticed that above a certain flow rate, Rayleigh streaming becomes less effective. Johansson et al. [92] also observed an increase in the mixing efficiency of liquids with different densities on applications of standing wave in microchannels.

## 3. Reactor Fabrication

Contrary to the extensive literature on the mechanisms of ultrasound, fewer details on the design, fabrication and characterization of ultrasonic flow reactors have been reported. This section will classify the reported reactors into categories, and then summarize their advantages and drawbacks. Characterization methods to assist the reactor design are introduced subsequently.

### 3.1. Reactor Design

Ultrasonic flow reactors usually consist of an ultrasonic transducer and a microfluidic device. The transducer, typically based on piezoelectric materials, converts alternating current into ultrasonic vibrations. It is normally actuated by a power amplifier driven with a sine wave from a signal generator. Based on the type of transducer used, ultrasonic reactors can be classified as piezoelectric plate based reactors or Langevin-type transducer based reactors.

#### 3.1.1. Piezoelectric Plate Based Reactor

**Figure 4:**
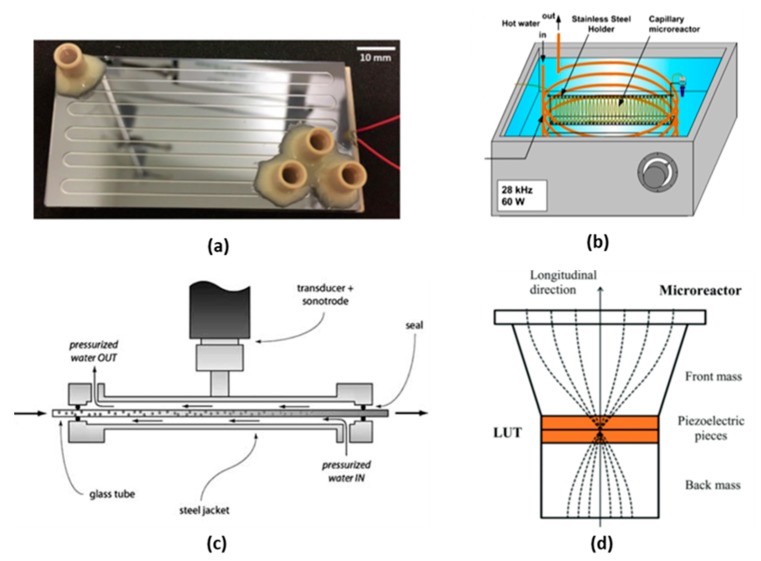
Representative examples of four categories of ultrasonic flow reactors. (**a**) Picture of a piezoelectric plate reactor developed by Dong et al., the reactor consists of a piezoelectric plate glued to the bottom of a silicon plate microreactor, reprinted with permission from [93], copyright Elsevier. (**b**) Capillary microreactor immersed in an ultrasonic bath, reprinted with permission from [97], copyright Elsevier. (**c**) Sketch of a Langevin-type transducer indirectly coupled reactor, reprinted with permission from [98], copyright Elsevier. (**d**) Sketch of a Langevin-type transducer directly coupled reactor, reprinted with permission from [34] copyright Royal Society of Chemistry.

Piezoelectric plate reactors can be built by directly coupling a piezoelectric plate to the surface of a microreactor. Often, the two parts are bonded together by epoxy glue [71,81]. In some cases, at low ultrasonic power, the two parts can be clamped together with the use of transmission grease to ensure good contact between the plate and the reactor [43,78]. Although the ultrasound transmission efficiency of this method might be lower compared to the use of epoxy glue, it allows disassembly, reuse and replacement of the two parts. A piezoelectric plate transducer can work under different resonance modes allowing multiple resonance frequencies, at which the reactor has a higher energy transfer efficiency. For example, the reactor developed by Dong et al. [93] consists of a piezoelectric plate with length, width and depth of 80 × 40 × 1.67 mm^3^ glued to the bottom of a silicon plate microreactor, see Figure 4a. The measured impedance curve shows that the reactor had several resonance peaks, between 20 kHz and 2 MHz, corresponding to different vibration modes with the main resonance peak of the thickness vibration mode located at 1.2 MHz. Consequently, piezoelectric plate reactors can operate at both low and high frequencies. Furthermore, they are versatile, simple to fabricate and easy to operate, making them the most widely used ultrasonic flow reactors in academia. Especially for acoustofluidic applications, this reactor is normally designed as a layered resonator, in which the thickness of the piezoelectric plate, reactor layer and cover plate match either a half or a quarter wavelength, resulting in highly efficient resonance vibrations in the thickness direction [81,94,95,96]. However, the load power is limited due to the tensile strength limitations of the piezoelectric material.

#### 3.1.2. Langevin-Type Transducer Based Reactor

For applications requiring relatively high ultrasonic powers, Langevin-type transducers are regarded as the most cost-effective choice, especially for low frequency ultrasound [99,100,101]. This transducer is made of piezoelectric ceramic rings clamped between a front and a back mass, which both serve to protect the delicate ceramic and prevent it from overheating by acting as a heat sink [59,96,102]. As the front mass is usually made of a light metal and the back mass a heavy metal, the ultrasound wave is mainly irradiated from the front surface. Sometimes, a sonotrode is connected to the front surface, in order to guide the ultrasound to the working material [103,104]. Langevin-type transducers typically dominate for applications where relatively large reactor volumes and thus high ultrasonic powers are required [105,106,107].

Based on the connection method between the transducer and microreactor, ultrasonic flow reactors can be divided into two categories, i.e., directly coupled and indirectly coupled. The former uses epoxy glue or a clamp to directly connect the transducer to the microreactor surface [34,107,108], while the latter utilizes a transmission medium (usually liquid) to transport ultrasound from the transducer to the microreactor [68,109,110,111]. The easiest way to construct an indirectly coupled reactor is by immersing a microreactor in a commercial ultrasound cleaning bath, under which several Langevin transducers are attached, see Figure 4b [97,112,113,114]. The drawback of such a setup is that the water in the bath is also cavitating, which dissipates most of the input ultrasound energy and thus only a small portion of energy reaches the microreactor. To overcome this problem, Hübner et al. [109] positioned the microreactor above the transducer in a stainless steel vessel filled with pressurized water (at about 4.5 bar), which acts simultaneously as heat and ultrasound transfer medium. Similarly, Freitas et al. [98] developed an ultrasonic flow-through cell consisting of a cylindrical steel jacket, in which a glass tube of 2 mm inner diameter for conveying the fluids was installed, see Figure 4c. A sonotrode fixed to a Langevin-type transducer was welded to the outside of the steel jacket to provide ultrasonic vibration. Through the space between the glass tube and the jacket, pressurized water (between 4.5 and 5.5 bar) was passed for sound conduction and temperature control. These indirect coupling methods have advantages of modularity and good temperature control [109], but also disadvantages of low energy transmission efficiency due to the attenuation in the transmission medium and reflection at the two liquid/solid interfaces [109].

Direct coupling is a more efficient way to transport ultrasound energy. Tseng et al. [107] directly bonded a glass plate microfluidic chip to the front face of a Langevin transducer with epoxy glue. A strong acoustic field was transferred into the microfluidic channel via the flexural lamb wave vibration of the glass plate, which is highly sensitive to the thickness, density, elastic properties and structure of the microreactor. Dong et al. [34] matched the structure of a Langevin transducer and a microreactor plate to form a half wavelength resonator in the longitudinal direction, where the antinode plane with highest sound intensity is located at the microreactor, see Figure 4d. This novel design not only generates a uniform and strong acoustic field density, but also maximizes the energy efficiency and lifespan of the transducer. Despite these advantages, direct coupling reduces flexibility and introduces difficulties regarding temperature control. As the transducers and microreactors are usually rigidly glued together, disconnecting and replacing them is normally not easy. Moreover, the heat generated by the ultrasonic transducer is directly transported into the microreactor, and in case of high input power, air cooling is even not sufficient to remove the large amount of heat [34,67,115]. One method to alleviate such temperature rise is to apply pulsed ultrasound, in order to reduce the power consumption. In some cases, it has been reported that applying ultrasound in a pulsed mode does not reduce the acoustic effect compared to continuous mode [116,117,118]. Delacour et al. [45] reported that applying ultrasound for 12.5% of the residence time was sufficient to prevent microchannel clogging for the synthesis of barium sulfate particles, while decreasing the temperature rise of the reactor from 7 to less than 1 °C after 5 reactor volumes. Dong et al. [93] found that applying ultrasound 37.5% per residence time produces a particle size distribution as narrow as that of continuous ultrasound for the synthesis of calcium carbonate particles. Another method to solve the temperature control issue is by combining direct and indirect coupling. John et al. [115] developed a hybrid contact reactor consisting of a Langevin transducer bolted to a mini-bath, in which PFA tubing was inserted, which was in contact with the transducer at several separate intervals, see Figure 7c. The intervals directly transported ultrasound from the transducer to the tubing, while the cooling water in the bath was used to both transmit ultrasound and control the temperature in the reactor. This hybrid system performed better than the indirectly coupled reactor (20–27% increase in yield) for a liquid–liquid extraction process.

Most of the ultrasonic flow reactors reported in literature can be classified into one of the above described reactor categories. The different designs applied in practice are described in the following section. It was found that piezoelectric plate reactors are mostly used at a laboratory scale because of their versatility and ease of fabrication. While Langevin-type reactors are more applied for both large and small scale applications, due to higher ultrasound energy transmission efficiencies and a wider operating range in terms of power. Remarkably, ultrasonic bath reactors are commonly used in organic synthesis, which is mostly due to their availability, operability and flexibility.

### 3.2. Reactor Characterization

Understanding the mechanisms behind low and high frequency ultrasound applied to confined channels is important for the design of ultrasonic micro- and milli-reactors [119]. For this reason, different characterization methods have been developed, which have mostly been applied to batch reactors. Table 1 aims to summarize the characterization methods. The objectives and equipment needed for those measurements are also described.

When working with ultrasonic devices, it is necessary to determine the resonance condition of the system. This information can be provided by impedance analyses [34,45,124], where the reactor impedance is measured as a function of actuation frequency, which indicates the anti-resonance and resonance frequencies of the reactor, mentioned in Section 3.1.

Low frequency ultrasound is associated with the collapse of cavitation bubbles, which results in a local increase of pressure and temperature. These primary effects can then also induce secondary effects, e.g., the formation of radicals. The characterization methods applicable to low frequency ultrasound aim to qualitatively or quantitatively measure these primary and secondary effects.

Hydrophones are able to quantitatively measure the acoustic pressure field distribution in a reactor cavity, which allows us to locate the nodes and antinodes of an acoustic wave, i.e., the most active parts, in terms of cavitation activity [76,77]. This method was used by Verhaagen et al. [121] to determine the position in an ultrasonic bath where their cavitation intensification bag (CIB) would be most effective. However, hydrophone measurements show some drawbacks, for instance it is not possible to directly obtain the acoustic pressure field in the reactor channel as the hydrophone probe diameter is usually larger than the channel diameter. The hydrophone probe could also disturb cavitation activity patterns, as it can act as a nucleation site for cavitation bubbles.

A second approach to obtain a qualitative distribution of the acoustic pressure field is through sonochemiluminescence. As mentioned earlier in Section 2.1, the collapse of cavitation bubbles leads to the formation of radicals, such as $HO^{.}$ and $H^{.}$, which can then react with chemicals. This method is based on the reaction between 3-aminophtalhydrazide, also called luminol, with $HO^{.}$ radicals to emit luminescence light. With this method, a visual representation of the cavitation zones in a reactor can be obtained by using a camera with long term exposure [76,119,120,121]. Using this method, Tandiono et al. [76] found that cavitation effects are more profound near a gas–liquid interface. This led to the introduction of a gaseous phase for a range of applications to improve cavitation phenomena, discussed in Section 4.

The cavitation activity in a reactor can also be quantified using chemical dosimetries. The most used chemical dosimetry is the Weissler reaction. This method is based on the degradation of potassium iodide into triiodure ions ($I_{3}^{-}$), due to the reaction with the radicals produced by ultrasound, which can be then quantified by ultraviolet spectrophotometry [119,121,122,123]. This method has been used by Pohl et al. [126] to compare the cavitation intensity in two reactors consisting of a sonotrode attached to either a conical or a cavitation reaction chamber. Results showed that cavitation intensity was higher in the cavitation reaction chamber than in the conical reactors, as zones without ultrasonic irradiation might be present in the latter. Similar dosimetries can be used to quantify cavitation activity. Fricke dosimetry is based on the oxidation of ${\mathrm{Fe}}^{2+}$ to ${\mathrm{Fe}}^{3+}$, whereas organic compounds produced by the reaction between salicylic acid and free radicals produced by ultrasound can be quantified by HPLC analysis. However, chemical dosimetries only allow the quantification of the overall cavitation activity in a given sonochemical reactor, the local cavitation activity cannot be quantified.

The collapse of cavitation bubbles will also result in an increase of the fluid temperature, and hence recording the sonochemical reactor temperature change enables the quantification of cavitation activity [119]. A temperature probe can be used to obtain the temperature field, which in turn reveals the location of cavitation zones. However, the violent collapse of cavitation bubbles might damage the temperature probe, and their size restricts their applicability in microfluidics. A non-invasive approach to determine a qualitative temperature distribution in a reactor is based on thermal imaging. John et al. [106] compared the temperature distribution inside a direct contact reactor and an interval-direct contact reactor using a thermal camera. For the interval contact reactor hot spots were observed at the intervals, whereas for the direct contact reactor hot spots were distributed on the entire surface of the micro-channel. Calorimetric measurements can be performed to determine the overall temperature increase in the fluidic channel, which in turn allows an estimation of the power density [30,45,122,123,127], according to

(2) $$P_{cal}=mc_{p}\frac{\Delta T}{\Delta t},$$

where m is the mass of the liquid medium, and $c_{p}$ is the specific heat capacity of the medium at constant pressure, where $c_{p}$ is considered constant in the measured temperature range.

While several experimental approaches exist to characterize sonochemical reactors, they are also associated with drawbacks, e.g., inserting temperature probes or hydrophones in a reactor might affect the cavitation activity distribution. As such, most experimental studies provide qualitative information, and in addition to them, numerical simulations can be used to optimize the ultrasonic field distribution inside reactors and to increase the understanding of the ultrasound phenomena. For this, experimental studies serve as validation for the numerical methods. The most investigated effect of ultrasound by numerical simulation is the acoustic pressure distribution, which can be predicted by solving the Helmholtz equation [104,120,125]. Rossi et al. [120] investigated the acoustic wave propagation and attenuation in a PMMA reactor by solving the Helmholtz equation:

(3) $$\nabla^{2}p_{a}+k_{m}^{2}p_{a}=0,$$

with $p_{a}$ the pressure amplitude and $k_{m}$ the complex wave number. The author defined a Dirichlet boundary condition at the wall and a pressure boundary condition at the source. Results showed that the transient cavitation zones could be predicted by numerical simulation. Other primary and secondary effect of low frequency can be investigated [125]. This work and those similar show that the numerical model, studied parameters and boundary conditions are linked and specific to the studied application and reactor design [125,127,128].

## 4. Applications

Single phase systems have, for a large part, been used in the detailed mechanism studies discussed in Section 2.1. The concepts and effects, such as mixing, observed in these systems have inspired the use of these mechanisms for multiphase applications. Increased reaction rates with the application of ultrasound can be readily found in literature [67,109], which is usually ascribed to effects such as streaming, increased interfacial area or the combination of both. On the other hand, the extent to which each of these effects contribute along with a detailed study of the mechanisms in multiphase systems, very little is known. This section contains the most relevant studies on the roles that ultrasonic mechanisms play in ultrasonic flow reactors along with several applications.

### 4.1. Gas–Liquid Systems

Dong et al. [38] characterized the mechanisms behind a 3–20-fold increase in mass transfer of a directly sonicated microreactor over that of unsonicated conditions. For gas–liquid Taylor flow in a microchannel, when the applied ultrasonic power is increased above certain threshold powers, gas bubbles in the channel start to oscillate in different modes. For each surface mode, the specific surface area can be described by the wavelength and amplitude of the oscillating interface, knowing the geometry of the bubble. For a bubble oscillating under the Faraday capillary mode, the specific surface area increases significantly from 30% to 160% with increasing power, however, the onset of this increase was limited by the channel size. As mentioned earlier for channels with a smaller cross-section, more power is required to overcome the threshold to initiate surface wave oscillations, due to the confinement effect [66], as explained in Section 2.1.

Cavitation microstreaming was also observed and characterized by streaming velocities measurements using streak photography for the same system under sonication. Two additional vortices associated with cavitation microstreaming were shown to interact with the regular Taylor flow pattern, which resulted in vigorous and dynamic streaming that increased with power. Using the Higbie penetration model [129], the mass transfer coefficient under sonication ($k_{L}^{\prime }$) could be described by the streaming velocity ($U_{A}$) at the gas–liquid interface:

(4) $$k_{L}^{\prime }=\sqrt{\frac{U_{S}+U_{A}}{U_{S}}}k_{L},$$

with $U_{S}$ the bubble’s slip velocity and $k_{L}$ the mass transfer coefficient in silent conditions. Mixing due to cavitation microstreaming would immediately enhance mass transfer from the onset of ultrasound, with a more profound effect on channels with larger diameters, since there is less recirculation to begin with. Whereas an increase in specific surface area would only occur in after a certain amount of applied power, especially for smaller channels. Eventually both enhancements, due to cavitation microstreaming and increased specific surface area, plateaus to within the same range for all the channels sizes with increasing power. For a gas–liquid Taylor system, Tandiono et al. [76] found that cavitation activity was more extensive pronounced near the gas–liquid interface compared to that of the rest of the liquid slug. From a sequence of high-speed images, it was seen that capillary waves at the interface would entrap small gas bubbles, these bubbles would then serve as nuclei for cavitation bubbles. With the introduction of a gas phase the effects of ultrasound can be enhanced significantly, a challenge that has proven difficult due to the confined space and reduced volume of microchannels. Zhao et al. [39] were able to utilize this for vanillin extraction, nitrogen gas was introduced to improve mass transfer even further over that of typical liquid–liquid extraction under sonication, which is discussed in the following subsection. When a third gaseous phase is introduced to a sonicated liquid–liquid system, surface wave oscillation as well as acoustic streaming at the gas–liquid interface enhances mass transfer in a similar way as for gas-liquid systems [39,130,131,132].

Navarro-Brull et al. [133] utilized ultrasound to improve liquid-gas dispersion throughout their micropacked-bed reactor, the authors suggest that the motion of particles with sonication reduces gas channeling through effectively fluidizing the packed bed. Results show a reduction in axial dispersion of two orders of magnitude with ultrasound.

### 4.2. Liquid–Liquid Systems

**Figure 5:**
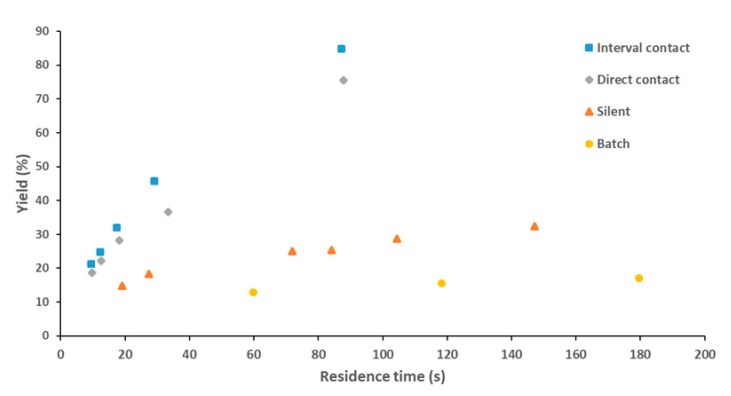
Reaction yield for the hydrolysis of *p*-nitrophenyl in a stirred batch reactor, unsonicated (silent) flow reactor, direct contact ultrasonic flow reactor and the five interval contact ultrasonic flow reactor, reproduced with permission from [106] and [108], copyright Elsevier.

When liquid–liquid extraction is carried out in a microchannel with the application of ultrasound the extraction efficiency is significantly increased [108,134]. Although the same phenomena can be found in both gas–liquid and liquid–liquid systems, the way these phenomena enhance mass transfer differ to a certain extent. When ultrasound is applied to a liquid–liquid system, cavitation bubbles emulsify the immiscible liquids, significantly increasing the surface area available for mass transfer. Although the exact mechanism of how cavitation leads to emulsification is still unclear, there is no doubt that it plays a key role. Zhao et al. [134] observed that as cavitation bubbles oscillate vigorously within a microchannel, they often shuttle through the interface of the two immiscible fluids carrying with them a small film of the organic phase (in this case 1-octane) into the aqueous phase (in this case water). The unstable cavitation bubble then breaks up this film to form smaller emulsion droplets within the aqueous slug. Stepišnik Perdih et al. [135] propose that when cavitation bubbles in the aqueous phase (in this case water) implode near the aqueous-organic interface (in this case sunflower oil), microjets propel water through the interface into the bulk of the organic phase. Thereafter, due to interface instability near the initial implosion, a small amount of the organic phase containing the dispersed aqueous phase separates from the bulk forming a droplet in the aqueous phase, which, once exposed to ultrasound, breaks into smaller droplets until small enough to be freely immersed in the aqueous phase forming an emulsion. The ultrasonic flow-through cell reactor, depicted in Figure 4c, was able to emulsify liquids under contamination free conditions, producing vegetable oil-in-water emulsions with Sauter diameters of 0.5 µm, as well as spherical particles, from a poly(lactic-co-glycolic acid) (PLGA) solution in dichloromethane, with volume mean diameters less than 0.5 µm [98]. Recently John et al. [106] were able to improve the performance of a liquid–liquid extraction process to their previous work [108] by focusing the applied ultrasound directly only at short intervals, as mentioned in Section 3.1. This would allow for the aqueous and organic phases, to periodically emulsify and then coalesce resulting in an increase of interfacial area improving mass transfer of the system. From Figure 5, a clear increase in extraction performance is observed when switching from batch to continuous flow, which is significantly increased with the application of ultrasound. Later the interval contact and hybrid rector was designed to improve performance, both showing similar and improved results [115].

### 4.3. Liquid–Solid Systems

When solid particles enter the system as a product or byproduct, particle deposition on the channel walls that can lead to channel fouling becomes a serious issue. Extensive research has been carried using ultrasound for organic and material synthesis as well as crystallization to prevent or at least mitigate this problem, with promising results [15,19,43,45,49,136,137,138,139]. Reports show that not only are reactors able to operate for significantly longer times without clogging or fouling, but smaller particles can be obtained with narrower size distributions along with increased reaction rates [45,49,93]. The mechanisms behind increased mixing have been discussed already in Section 2.1 and differ little to none with the presence of solid particles, however, the influence of these mechanisms on the solid particles themselves will be discussed in this subsection.

Though the mechanisms differ, both high and low frequency ultrasound has proven successful in reducing particle size and help prevent clogging. The cavitation effect of low frequency ultrasound has also aided in enhancing process conditions. Yang et al. [140] utilized low frequency ultrasound to synthesize zinc oxide quantum dots with better size control and increased quantum yield up to 64%. Sebastian et al. [141] observed that under influence of low frequency ultrasound, synthesis of gold-palladium dumbbell-like nanostructures could be carried out at milder temperature of 25 °C instead of 100 °C, also the residence time was reduced from 5 to 2 min and clogging was avoided. It was speculated that the localized high temperature, due to transient acoustic cavitation, proved beneficial at milder reaction conditions. For the case of low frequency ultrasound, not only does acoustic cavitation promote mixing and reduce particle deposition on channel walls, but it also leads to particle and agglomerate breakup. Resulting in smaller particles with narrower size distributions as mentioned in Section 3.1 [45,93].

Similar results can be achieved with high frequency ultrasound. Reactors are able to operate for longer periods of time due to particle focusing to the center of the channel, which also narrows the velocity distribution leading to shorter growth times and a monomodal distribution [49]. There are reports where high frequency ultrasound has led to a reduction in particle size [49,93]. Dong et al. [49] reduced both the average particle size and the distribution with high frequency ultrasound. Here focused particles are prevented from attaching to the channel walls, where they would grow to either clog the channel or detach as agglomerates. However, the high concentration of particles at the nodal plane can lead to particle agglomeration. In an attempt to avoid this, Dong et al. [93] combined both high and low frequency ultrasound in periodic intervals to successfully reduce the amount of agglomerates from 21% at high frequency ultrasound to 4.5–6.7% in the reactor effluent, along with a significant reduction in power consumption (50–75%) when compared to the continuous application of low frequency ultrasound.

**Figure 6:**
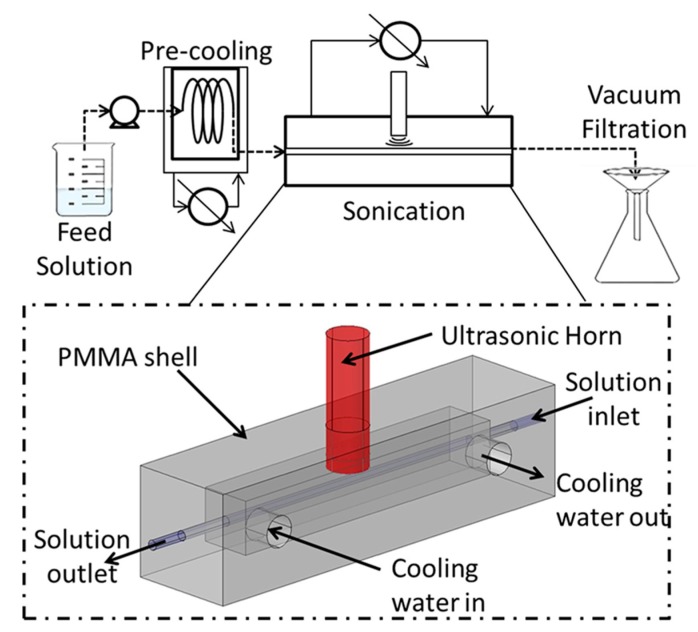
Schematic of the capillary sonocrystallizer setup used by Valitov et al. to study the effect of acoustic streaming on crystallization, reprinted with permission from [105], copyright Elsevier. The feed solution was pumped through the pre-cooling section to reach supersaturation and underwent sonocrystallization in the sonication section.

Ultrasound assisted crystallization has long been a topic of interest, not only can crystal sizes and clogging be reduced with ultrasound, as in the case for organic and material synthesis, but there is also a reduction in induction time and metastable zone width [105,128,137,142]. Rossi et al. [120] observed that, in their droplet-based microfluidic crystallizer, crystal nuclei were generated easier at a lower supersaturation with sonication, compared to silent conditions. Under conditions where primary nucleation would not occur spontaneously, Hussain et al. [142] showed that sonication can lead to nucleation without the addition of seeding particles. Speculation suggests that a cavitation bubble can either induce nucleation through expansion, which would lead to the evaporation of the surrounding liquid, cooling down a small zone, or reduce the Gibbs free energy enough to form a stable nucleus. Acoustic cavitation can also lead to particle breakup, producing nuclei for secondary nucleation to occur speeding up the crystallization process. Valitov et al. [105] placed an ultrasonic horn closed to the capillary tube to study the effect of acoustic streaming on sonocrystallization as seen in Figure 6. They observed that an increase in acoustic streaming led to backmixing and lower local supersaturation, which resulted in smaller crystal sizes.

Table 2 summarizes the different processes studied in ultrasonic flow reactors. The reported effects of ultrasound on the specific applications are provided, as well as a short description of the reactor, including the reactor category as mentioned in Section 3.1, along with the working frequency, applied power/voltage and reactor dimensions. The scale and/or scale up strategy, as described in Section 5, is also mentioned.

## 5. Scale-Up of Ultrasound Reactors

In the previous parts of this review, the combination of ultrasound and microreactors has been described at a laboratory scale. In this part, the scalability approach of such reactors will be investigated. As for small scale reactors the main challenge is the distribution of acoustic field. The choice of the scalability approach is highly dependent on the expected effect of ultrasound on a specific application.

Concerning the scalability of microreactors alone, two main strategies have been developed over the last decade [151,152]. The first method, known as scaling out, consists of increasing the characteristic size of the channels. The second method, numbering up, is achieved by running several identical units in parallel. Both methods are effective to a different extent, which mostly depends on the application and reactor design. Since these reactors are able to run continuously, they have an intrinsic advantage when it comes to meeting industrial production demands. Not only do continuous reactors allow for better control of the final product quality, but they are also able to reach higher production rates compared to batch reactors [153]. As mentioned earlier, Table 2 includes the different reactors described in this part and highlights the scale-up strategies.

**Figure 7:**
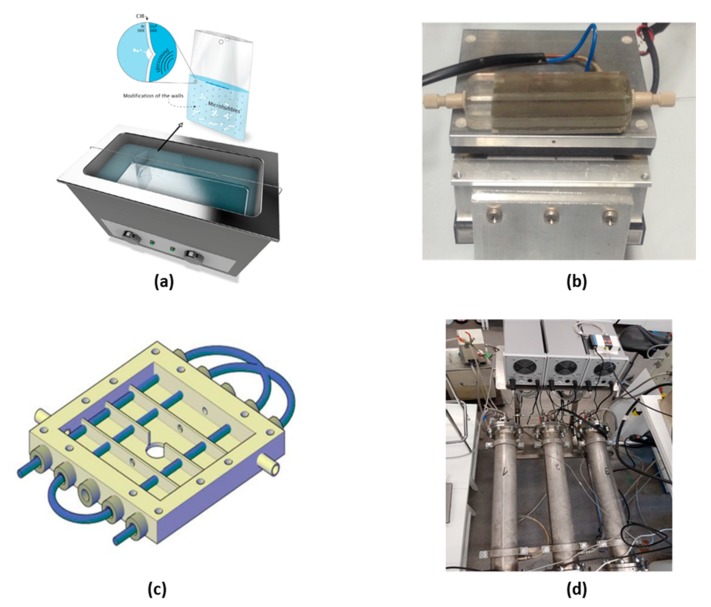
Examples of scaled-up reactor designs: (**a**) cavitation intensification bag immersed in an ultrasonic bath (numbering up), reprinted with permission from [143], copyright Elsevier. (**b**) Scale out strategy for sonocrystallization, reactor consisting of a piezoelectric plate attached to a glass capillary, reprinted with permission from [128], copyright ACS publications. (**c**) Scale out strategy for liquid–liquid extraction, reactor consisting of PFA tubing immersed in an hybrid ultrasonic reactor, reprinted with permission from [115], copyright Elsevier. (**d**) Combination of scale out and numbering up strategies for a sonocrystallization process, reactor consisting of a sonotrode and a reactor wrapped as a helix around the sonotrode, reprinted with permission from the authors [149].

The main parameter limiting the number of applications of ultrasonic milli-reactors at industrial scale is the acoustic pressure field distribution in the liquid medium. Verhaagen et al. [121,154] developed a scaled-up sonochemical microreactor with increased reproducibility and efficiency and was also able to clearly observe cavitation phenomena. The numbering up strategy applied by Van Zwieten et al. [143] was carried out by immersing several cavitation intensification bags in an ultrasonic bath for the formation of a hexadecane and SDS aqueous emulsion, see Figure 7a, to obtain droplets diameter of 0.2 µm. To characterize the cavitation activity, three methods have been used: sonochemiluminescence of luminol, hydrophone measurements and terephthalic acid dosimetry. As mentioned previously these methods allowed for a reactor configuration that made effective use of the cavitation phenomenon, an essential aspect of scaled-up reactor designs and operation.

Another strategy consists of enlarging channel size. This method has been used by Jamshidi et al. [128] for the design of an ultrasonic millifluidic device consisting of glass capillary channel with a cross-section of 2 × 5 mm^2^ for the crystallization of adipic acid, see Figure 7b. This reactor has been designed with the use of numerical simulations to obtain the acoustic pressure distribution throughout the reactor. Smaller crystal sizes were obtained compared to conventional batch reactors. John et al. [115] also applied the same strategy for the design of the interval contact reactor, see Figure 7c, for liquid–liquid extraction, discussed in Section 3.1. The study focused on the effect of increasing the channel diameter size from 0.8 to 2 mm on the performance of p-nitrophenylacetate hydrolysis, with a better relative performance obtained with the 2 mm tubing.

Depending on the application, the combination of the two scale up strategies previously described can be used. Gallaher et al. [147] developed a sonocrystallization reactor device that consists of tubing with piezoelectric ring attached to it and oil flow is used to control temperature. Tubing diameter and number of reactor units can be adjusted depending on the production demand. Koiranen et al. [148] also combined the two strategies. The authors designed a reactor unit that consisted of a sonotrode with tubing wrapped as a helix, see Figure 7d. This method ensured a direct contact as described in Section 3.1. Temperature control is achieved by using a heat transfer fluid flow. Numbering up was performed by running three units in parallel. Ezeanovi et al. [149] were able to successfully apply this scaled-up reactor for a crystallization process, preventing clogging and promoting nucleation.

As mentioned before, another approach to scale up is based on working under continuous conditions with larger vessels [155,156,157]. Nickel and Neis [155] developed a 29 L pilot scale sonochemical reactor, equipped with five 20 kHz transducers for studying the disintegration of biosolids. A second continuous large scale reactor example was used by Gondrexon et al. [156] for the degradation of an aqueous solution of pentachlorophenol. This reactor was designed as a three stage distillation column with a 500 kHz transducer attached below each stage.

As a conclusion, the choice of the scale-up strategy not only depends on the understanding of the ultrasonic mechanisms but also on the coupling between ultrasonic device and reactor. In fact, direct coupling between piezoelectric devices and reactors, as described in Section 3.1, might be more suitable for numbering up whereas coupling of Langevin transducer with tubing, as the hybrid reactor developed by John et al. [115] might be for scaling out. Combining the two scale-up strategies seems promising to fulfill the requirements of the chemical industry, as small scale reactors offer better control of the final product properties with increased production rates. Despite the need for further studies, currently on-going efforts on numerical characterization of the scalability of such reactors show promise to increase the range of applications.

## 6. Conclusions

Small scale flow reactors have long been regarded as the way forward for various chemical processes, especially when considering switching from batch to continuous. Integrating ultrasound has brought this technology one step closer into making this a realization by, not only mitigating the inherent problems of microreactors, but also increasing their versatility and broadening applicability through the different mechanisms associated with ultrasound. Detailed studies of these mechanisms make it possible to have a broader outlook on different applications, having already shown promise for several. From lab to pilot scale ultrasonic flow reactors have proven to outperform conventional equipment, however, scaling of these reactors to meet the output of their conventional counterparts is still a work in progress. Problems regarding temperature control, uniform ultrasound distribution and the low energy transfer efficiencies are currently being investigated. Design techniques for larger scales are making it possible, for instance, to fabricate reactors in such a way to distribute the ultrasound energy where required, reducing the power consumption and the need for excessive cooling. Whereas reactor characterization identifies the most active zones in reactors, making it possible to utilize ultrasonic effects to a larger extent. Both these methods have also been used to promote these effects to such an extent that there are various scaled designs currently being tested for industrial applications.

## Figures and Tables

**Table 1:** Summary of major characterization methods and the corresponding objectives and procedures.

Method	Type of Method	Objectives	Materials	Reference
Sonochemiluminescence of luminol	Experimental, Chemical, Qualitative	Observation of cavitation activity distribution	Aqueous solution of luminol and sodium hydroxide.	[76,120,121]
Dosimetries: salicylic acid, Fricke, Weissler, terephthalic acid	Experimental, Chemical, Qualitative	General cavitation activity measurement, cavitation yield	Analysis method: spectrophotometry, HPLC analysis.	[119,121,122]
Hydrophone measurement	Experimental, Physical, Quantitative	Acoustic pressure mapping. Observation of standing waves.	Hydrophone probe, oscilloscope.	[76,119,121]
Temperature mapping	Experimental, Physical, Qualitative	Temperature mapping to observe hot spots.	Thermal camera.	[106]
Calorimetric measurement	Experimental, Physical, Quantitative	Temperature rise measurements. Estimation of power density.	Temperature probe.	[30,122,123]
Impedance measurement	Experimental, Physical, Quantitative	Resonance conditions: resonance and anti-resonance frequency.	Impedance analyzer	[34,45,124]
Pressure acoustic mapping	Numerical, Quantitative	Helmholtz equation	Numerical simulation software	[119,120,125]
Simulation of primary and secondary effect	Numerical, Quantitative	Temperature, bubble yield	Numerical simulation software	[119,125]

**Table 2:** Summary of the different applications and process enhancement in ultrasonic flow reactors.

**Processes**	**Ultrasound Effect and Application**	**Reactor Description**	**Reactor Scale**	**Reference**
**Liquid** **(single phase)**	Cavitation to improve mixing of dye and water	Langevin-type transducer reactor, direct coupling 20 kHz, 10–30 W Silicon microreactor: channel size 1 × 1 mm^2^, 0.5 × 0.5 mm^2^ and 0.5 × 0.25 mm^2^	Laboratory scale	[24]
	Cavitation to improve mixing of glycerol and water	Piezoelectric plate reactor 38.9 kHz, 160 Vpp PDMS microreactor: channel size 0.24 × 0.15 mm^2^	Laboratory scale	[75]
	Ultrasound assisted nitration of toluene	Langevin-type transducer reactor, hybrid contact 21 kHz, 50 W Stainless steel capillary: inner diameter 0.6–1 mm	Laboratory scale	[67]
**Gas/liquid**	Cavitation and surface wave oscillation to improve gas-liquid mass transfer for carbon dioxide absorption	Langevin-type transducer reactor, direct coupling 20 kHz, 10–50 W Silicon microreactor: channel size 1 × 1 mm^2^, 0.5 × 0.5 mm^2^ and 0.5 × 0.25 mm^2^	Laboratory scale	[38]
**Gas/liquid/solid**	Sonication to partially fluidize a micro-packed-bed reactor to reduce gas-channeling	Langevin-type transducer reactor, direct coupling 38 kHz, 20 W Micropacked-bed reactor: inner diameter 3.175 mm, diameter of packed beads 0.2 mm	Laboratory scale	[133]
**Liquid/liquid**	Surface wave oscillation with the introduction of a gas phase to improve liquid-liquid extraction	Langevin-type transducer reactor, direct coupling 20 kHz, 5–30 W Silicon microreactor: channel size 1 × 1 mm^2^	Laboratory scale	[39]
		piezoelectric plate reactor 1–100 kHz, 10–20 Vpp PDMS microreactor: channel size 0.2 × 0.05 mm^2^	Laboratory scale	[130]
	Ultrasound assisted reactive extraction of p-nitrophenylacetate	Langevin-type transducer reactor, direct contact 20.3 kHz, 20–29 W PFA Capillary: inner diameter 0.8 mm	Laboratory scale	[108]
		Langevin-type transducer reactor, hybrid contact 20–65 kHz, 20 W PFA Capillary: inner diameter 0.8–2 mm	Scale up strategy: scale out	[115]
	Cavitation to emulsify and improve mixing for the extraction of rhodamine B from water to 1-octanol	Langevin-type transducer reactor, direct coupling 20 kHz, 10–30 W Silicon microreactor: channel size 1 × 1 mm^2^ and 0.5 × 0.5 mm^2^	Laboratory scale	[134]
	Ultrasound for oil-water emulsion and PLGA nanoparticle synthesis	Langevin-type transducer reactor, indirect coupling 24 kHz, 17–32 W Glass tube: inner diameter 2 mm	Laboratory scale	[98]
	Cavitation to enhance emulsification of hexadecane in SDS aqueous emulsion	Ultrasonic bath reactor 37 and 80 kHz, around 180 W Cavitation intensification bag: plastic bag with pits	Laboratory and large scale Scale up strategy: numbering up	[143]
**Processes**	**Ultrasound Effect and Application**	**Reactor Description**	**Reactor Scale**	**Reference**
**Liquid/solid** **Material synthesis**	Cavitation leading to milder reaction conditions applied to Dumbbell shaped Au-Pd nanoparticle synthesis	Piezoelectric plate reactor 40 kHz, 30 W Silicon microreactor: square channel 0.4 × 0.4 mm^2^	Laboratory scale	[141]
	Cavitation to prevent of clogging for AgCl nanoparticle synthesis	Ultrasonic bath reactor 40 kHz, power not mentioned PTFE Tube: inner diameter 1 and 2 mm	Laboratory scale	[144]
	Cavitation to change structure of ZnO quantum dots due to high energy hotspots	Ultrasonic bath reactor 53 kHz, 72–180 W PTFE Tube: inner diameter 0.8 mm	Laboratory scale	[140]
	Cavitation to promote uniform particle shape and size, improved crystal quality applied to precipitation of hydroxyapatite.	Reactor type 1: ultrasonic bath reactor 40 kHz, 4–8 W Teflon Tube: inner diameter 1.02 mm Reactor type 2: piezoelectric plate reactor 50 kHz, 30 W Teflon microreactor: channel width 0.6 mm	Laboratory scale	[44]
	Cavitation for clogging prevention, particle size control applied to barium sulfate precipitation	Langevin-type transducer reactor, direct coupling 21–46 kHz, 11–23 W Silicon microreactor: square channel 0.6 × 0.6 mm^2^	Laboratory scale	[45]
	Acoustophoresis for clogging prevention, particle size control applied to particle synthesis	Piezoelectric plate reactor 1.21 MHz, 0.3–3.3 W Silicon microreactor: square channel 0.6 × 0.6 mm^2^	Laboratory scale	[49]
	Combining cavitation and acoustophoresis for particle synthesis	Piezoelectric plate reactor 61.7 kHz (8 W) and 1.21 MHz (1.6 W), pulse and switch mode Silicon microreactor: square channel 0.6 × 0.6 mm^2^	Laboratory scale	[93]
**Liquid/solid** **Organic synthesis**	Cavitation for clogging prevention applied to C–N cross coupling reaction	Ultrasonic bath reactor 41.5 kHz, power not mentioned PFA tube: inner diameter 1.01 mm	Laboratory scale	[113]
		Ultrasonic bath reactor 41.5 kHz, power not mentioned PFA tube: inner diameter 0.5 and 1 mm	Laboratory scale	[15]
		Piezoelectric plate reactor 50 kHz, 30 W Teflon microreactor: channel width 0.6 mm	Laboratory scale	[43]
	Cavitation for clogging prevention applied to KMnO4 oxidation	Ultrasonic bath reactor 44 kHz, pulsed (5 s every minute), power not mentioned PFA tube: inner diameter 0.5 mm	Laboratory scale	[145]
	Cavitation for clogging prevention applied to photodimerization of maleic anhydride	Ultrasonic bath reactor 39 kHz, 100 W FEP tube: inner diameter 0.5–1.6 mm	Laboratory scale	[112]
	Cavitation for clogging prevention applied to arylation of aryl bromides	Ultrasonic bath reactor 40 kHz, 150 W Capillary coil: inner diameter 0.53 mm	Laboratory scale	[146]
**Liquid/solid** **Sonocrystallization**	Enhanced nucleation with ultrasound for adipic acid crystallization	Langevin-type transducer reactor, indirect coupling 20 kHz, 750 W, Amplitude 21% PFA Capillary: inner diameter 1 mm	Laboratory scale	[120]
	Enhanced anti-solvent mixing, reduced induction times and anti-solvent crystallization at a lower supersaturation with ultrasound for acetyl salicylic acid crystallization	Langevin-type transducer reactor, hybrid contact 42 kHz, 7–24 W PFA Capillary: inner diameter 2 mm	Laboratory scale	[142]
	Increased nucleation rate and smaller crystals size with pulsed ultrasound for adipic acid crystallization	Piezoelectric plate reactor 42–1090 kHz, pulsed, 400 mVpp, duty cycle 1%–7% Glass milli-reactor: channel 2 × 5 mm^2^	Scale up strategy: micro to milliscale	[128]
	Backmixing lead to lower yield, smaller crystal size with ultrasound	Langevin-type transducer reactor, indirect coupling 20 kHz, 750 W, amplitude 21% FEP Capillary: diameter 1.55 and 3.2 mm	Laboratory scale	[105]
	Cavitation for clogging prevention applied to crystallization processes (Patent)	Piezoelectric plate reactor Piezoelectric ring attached to tubing with adaptable diameter	Scale up strategy: micro to milliscale and parallel numbering-up	[147]
		Langevin-type transducer reactor, direct coupling Reactor wrapped as a helix around a sonotrode	Scale up strategy: micro to milliscale and parallel numbering up	[148,149]
		Langevin-type transducer reactor, indirect coupling Reactor wrapped as a helix and immersed in a jacketed beaker for temperature control. Ultrasonic transducer attached to the bottom of the beaker	Scale up strategy: micro to milliscale	[150]
